# Effect of a Strawberry and Spinach Dietary Supplement on Spatial Learning in Early and Late Middle-Aged Female Rats

**DOI:** 10.3390/antiox8010001

**Published:** 2018-12-20

**Authors:** Paula M. Millin, Gina T. Rickert

**Affiliations:** 1 Department of Psychology, Kenyon College, Gambier, OH 43022, USA; 2 Chicago College of Osteopathic Medicine, Midwestern University, Chicago, IL 60515, USA; grickert39@midwestern.edu

**Keywords:** aging, antioxidant, flavonoid, mitochondrial free radical theory of aging, Morris Water Maze, phytochemicals, vitamin C, vitamin E, spatial learning

## Abstract

The present experiment sought to determine the effect of an eight-week, high antioxidant, whole-foods dietary supplement on Morris Water Maze performance in early and late middle-aged female rats. To improve ecological validity over past experimental studies, rats in the current study received antioxidants by consuming freeze-dried organic strawberries and spinach rather than by being given food extracts or antioxidant injections. Latency and path length measures both indicated that late middle-aged rats fed the high antioxidant diet performed on a par with the younger animals earlier in training than their standard diet counterparts (*p* < 0.05). Superior performance was not due to improved fitness in the antioxidant-supplemented rats. Thus, our model showed that a high antioxidant diet of relatively short duration mitigated the mild cognitive decline that was seen in control animals during the developmental period of late middle-age. The current results offer support for the promising role of dietary antioxidants in maintaining cognitive health in normal aging and extend past findings to females, who have been relatively neglected in experimental investigations. Moreover, the current model suggests that the period of transition from early to late middle age is a promising target for dietary intervention in healthy adults.

## 1. Introduction

As advances in medicine, along with other factors, continue to increase human longevity [1] (p. 10), scientists are increasingly concerned with understanding not only disease-related changes in cognitive and affective functioning that increase with advancing age, but also in changes attributable to normal aging processes. Although individual differences can be large [2], generally speaking, both human and nonhuman animals show age-related declines in spatial and nonspatial learning and memory [3,4,5]. The exact age of onset of decline is debatable. Methodological variables, such as, in the case of human experiments, whether the design was cross-sectional or longitudinal and whether practice effects were controlled for [3], as well as contextual factors, such as goals and motivation [6], may account for the divergent estimates produced by experimental research.

Although numerous physiological processes likely contribute to cognitive decline in aging mammals, a general theory of aging first proposed by Harman in 1956, and later updated in 1972, The Mitochondrial Free Radical Theory of Aging (MFRTA), generated much empirical work in the area [7,8]. MFRTA states that the aging process, and ultimately death, results in part from the accumulation of mitochondrial damage caused by free radicals, which are cells that have lost a critical molecule during the oxidation process, making them unstable. The free radicals replace their lost molecule by taking one from a neighboring cell, damaging the DNA of that cell and perpetuating the cycle. Interest in the contribution of oxidative stress in disease processes ranging from depression and anxiety to cancer and Alzheimer’s disease (AD) soon followed [9]. Although evidence for oxidative damage as a major determinant of longevity is correlative and no longer universally accepted [10], oxidative stress is strongly implicated in the early pathogenesis of AD [11,12,13]. Likewise, there is abundant evidence that even in the absence of disease processes the aging brain is marked by increases in oxidative stress damage, especially in the hippocampus, a medial-temporal lobe structure that is fundamental to the formation of new long-term memories and to spatial recognition and navigation [5,13,14,15]. Moreover, there exists a positive correlation between oxidative stress in the hippocampus and learning impairment in aged rats [16,17], which made the accumulation of oxidative stress an attractive candidate for the cause of cognitive decline with aging. 

Although not a ubiquitous finding, some studies reported a loss of antioxidant defenses with age [18,19], suggesting, together with the aforementioned findings, that supplementing an organism’s diet with antioxidants, chemicals that donate electrons to free radicals without themselves becoming scavengers, might mitigate age-associated, as well as experimentally-induced, cognitive decline. Indeed, there are numerous reports of experimental rodent models showing positive effects of various antioxidant supplements [20,21,22,23,24,25]. For example, irradiation-induced deficits in Morris Water Maze (MWM) performance in young male rats were ameliorated by dietary supplementation with 2% strawberry or blueberry extract [26]. The Morris Water Maze is a forced swimming task that requires rats to use extra-maze visual cues to recall the location of an invisible platform that is submerged just under the surface of opaque water in order to find and mount the platform and escape from the water. The spatial nature of the task makes it strongly hippocampal-dependent and therefore a useful test for assessing cognitive decline in aging. In another study, antioxidant supplements comprised of strawberry extract, spinach extract, or Vitamin E given to male rats for eight months beginning at six months of age retarded age-related declines in both neuronal (e.g., signal transduction) and cognitive (e.g., MWM) function [27]. Moreover, the same antioxidant regimen was effective in reversing age-related deficits in 19-month-old male rats [28]. Even a short-term (eight weeks) antioxidant supplementation has been reported to reverse age-induced deficits in a simple straight alley motor learning task in male rats [29].

While most experimental studies on the effects of antioxidants have utilized male subjects, a small number have examined females. A 2004 study demonstrated that performance on MWM improved, antioxidant enzyme activity in the brain increased, and lipid peroxidation in the prefrontal cortex, striatum, & hippocampus reduced in 24-month-old ovariectomized female rats who were injected daily for three weeks with either deprenyl (which reduces free radical production and increases antioxidant enzymes), β-estradiol, or a combination of the two [30]. In 2005, a group reported that pretreatment for 30 days with injectable vitamins E & C prevented ovariectomy-induced memory deficits on MWM in young female rats [31]. Xu et al. (2009) reported that a seven-week supplementation of antioxidants derived from the lotus seed pod extract improved MWM performance in 18 month-old cognitively impaired female rats [32]. The current study sought to extend existing findings by examining the effects of a relatively short (eight weeks) real-foods antioxidant-rich dietary supplement consisting of freeze dried organic strawberries and spinach on cognitive performance in intact aging female rats. We believe using real foods lends ecological validity to the results and takes into consideration the finding that large megadoses of antioxidants, as is often found in dietary supplements, can have toxic (and even lethal) effects [33]. Strawberries and spinach were chosen from among the many antioxidant-rich foods because of their high flavonoid content, which recent research has suggested may be of special importance in alleviating cognitive impairments associated with aging [25] and because of their effectiveness in alleviating deficits in spatial learning in prior studies with male rats across various stages of the lifespan [26,27,28]. They are also readily available in freeze-dried form and highly palatable. Moreover, our use of intact, typically-developing (aging) rats, as opposed to young ovariectomized females, allows us to see the effects of antioxidants on general, age-related changes in learning and memory in females, rather than deficits related specifically to the loss of estrogen in the absence of other, more global age-related changes. The choice to intervene during early and late middle age stemmed from the desire to avoid studying organisms when they were either too young to show signs of cognitive decline (making it impossible to measure any effect of the dietary supplement) or so old that they were likely to have begun to experience the onset of neurodegenerative disease processes. Indeed, according to one review, failure to investigate the effects of antioxidants at the appropriate stage of development may contribute to the mixed success of antioxidant supplementation in human clinical trials [34]. We hope we have created a model to study the potential benefits of consuming high levels of dietary antioxidants during middle to late adulthood, when negative cognitive changes are just beginning to emerge. To that end, 12- and 16-month-old female rats were fed either a standard diet or a standard diet plus a real-foods dietary supplement of freeze dried organic strawberries and spinach rich in Vitamins C, E, & K, flavonoids and carotenoids for eight weeks prior to assessing performance on the MWM. 

## 2. Materials and Methods

### 2.1. Subjects

Forty-two adult female Long-Evans Hooded rats, bred at Kenyon College (derived from breeders purchased from Hilltop Laboratories, IN), were housed in pairs in large opaque plastic tubs (46 × 31 cm) lined with 5 cm of corn cob bedding. Twenty of the rats were 12 months old at the beginning of the experiment and twenty-two rats were 16 months of age at the beginning of the experiment. The colony room was maintained between 70 to 73 °F with a 12:12 h light:dark cycle (lights on at 7:00 a.m.). Cage-mates were separated in their home cages by a clear Plexiglas divider for approximately 10 h/day so that we could ensure that animals on the high antioxidant diet consumed all of their dietary supplement. Water and standard lab chow were available ad libitum in all conditions. Each rat was handled for 5 min per day for five consecutive days prior to beginning the behavioral assay portion of the experiment. All training and testing occurred during the lighted portion of the photocyle and at approximately the same time each day. All procedures, which followed the standards of the National Institutes of Health guide for the care and use of Laboratory Animals, were approved by the Kenyon College Institutional Animal Care and Use Committee on 12/17/2012.

### 2.2. Diet

Depending on group assignment (described below), animals were fed either a diet of standard Laboratory Rodent Diet ® 5001 rodent feed (manufactured by LabDiet®; purchased from Cincinnati Lab Supply, Inc., Cincinnati, OH, USA) or Laboratory Rodent Diet ® 5001 rodent feed plus a high antioxidant food supplement consisting of organic freeze-dried strawberries (3.03 g/kg body weight) and organic freeze-dried spinach (4.93 g/kg body weight; both purchased from Nuts.com). This supplement was measured to provide 91 mmol Trolox equivalent antioxidant capacity per kg, as well as high flavonoid and carotenoid levels (see Giampieri, Alvarez-Suarez, & Battino, 2014, [35] for review of strawberries’ nutritional composition; for review of the nutritional composition of spinach, see Ismail, Marjan, & Foong, 2004, [36]). The supplement was given once per day (at approximately 1:00 p.m. Eastern Standard Time), and rats were kept separated from their cage mate by a Plexiglas partition in the home cage until the supplemental food was completely consumed (approximately 10 h per day). Animals in both dietary conditions had unlimited access to lab chow and water at all times, including when separated by the partition. “Early middle-age” (EMA) animals (*n* = 20) in the “high antioxidant diet” (HAD) condition (Group EMA-HAD; *n* = 10) began receiving the dietary supplement at 12 months of age and continued it for eight weeks, at which time testing in the MWM began (at 14 months of age). “Late middle-age” (LMA) animals (*n* = 22) in the high antioxidant diet condition (Group LMA-HAD; *n* = 11) began the dietary supplement at 16 months of age and continued it for eight weeks, at which time testing in the MWM began (at 18 months of age). Age-matched rats receiving the control diet—groups EMA-Control (*n* = 10) and LMA-Control (*n* = 11)—remained on a standard diet with no supplementation during this period, but were also separated by a partition for 10 h/day to control for social housing effects. 

### 2.3. Apparatus & Procedure

#### Morris Water Maze

The Morris Water Maze is a commonly employed measure of spatial hippocampal-dependent memory. It was chosen because prior research has shown that the hippocampus is sensitive to oxidative stress and because hippocampal-dependent memory decline is often the first sign of memory problems in aging organisms. Water maze training was conducted in a round, black, fiberglass pool (173 cm diameter × 46 cm high; manufactured by Custom Fountains, Inc. Mason, OH, USA). Water was maintained at a depth of approximately 31 cm and temperature was maintained at 26 ± 3 °C. The water was made opaque by the addition of nontoxic white tempura poster paint (Crayola LLC., Easton, PA, USA) and an escape platform was placed approximately 2.5 cm below the water surface so that it was not visible to the animal but was high enough that the animal could not swim over it without bumping into it. The pool was located in a small room (2.1 m × 3 m) that had three white walls and one black wall (created by a black hanging curtain). Experimenters stood behind the black curtain during trials, making them invisible to the animal. Ninety-one centimeters above the floor on each of the three white walls hung large (61 cm × 91 cm) highly contrasting geometric shapes that were clearly visible to the animals from the water maze. A video camera was mounted directly above the center of the maze and all trials were recorded. The overhead fluorescent house lights were turned off (to reduce glare on the pool surface). Light was provided by two reflective aluminum clamp lights, each containing one 60-watt standard white light bulb. The lights were positioned in opposite corners of the room and pointed upward to produce diffuse light. Behind the black curtain was a counter holding a computer on which the experimenter could observe the animal’s real-time performance. Latency to mount the platform was recorded both manually (with a stop watch) and by SMART 3.0 computer software (Panlab, Harvard Apparatus, Barcelona, Spain). The software also collected other measures, including swimming speed and path length. 

Each animal received training on four consecutive days (days 1–4). During each training session animals received four trials, one beginning from each imaginary compass point on the pool (N, S, W, & E) in a randomly assigned order with approximately 5 (± 3) min intervals between trials. A trial involved placing the rat in the maze facing the pool wall and recording its latency (along with other measures) to mount the submerged platform. If an animal did not mount the platform within 60 s it was picked up and placed on it for 10 s, after which it was removed from the maze and returned to a holding cage outside of the experimental room. Each holding cage was made of clear polycarbonate and had a towel covering the bottom to absorb water. To help animals maintain normal body temperature, the holding cages were placed on top of heating pads and under heat lamps positioned 1m above the cages. After the final training trial on day 4, each animal received a probe trial, in which it was returned to the pool with the escape platform removed. Time spent in each quadrant, as well as swimming speed was recorded. Path length (recorded in inches and converted to centimeters) and swimming speed were also analyzed to confirm that differences in latency were not attributable to differences in nonspecific effects of the diet, such as motivation or fitness. The independent variables were day and condition. For the analyses, which were conducted using IBM SPSS statistics software, mean latency, path length, or swimming speed represent the average of the four daily trials. Critical alpha level was 0.05 for all omnibus analyses, although marginally significant differences (<10) are also reported. Partial eta-squared effect sizes are also reported where appropriate.

## 3. Results

### 3.1. Latency

A 4 × 4 mixed design analysis of variance (ANOVA) examining mean latency to find the platform by day and condition revealed significant main effects of day, *F*(3, 114) = 162.67, *p* = 0.000, *ηp*^2^ = 0.81, and condition, *F*(3, 38) = 3.21, *p* = 0.034, *ηp*^2^ = 0.20, and a significant interaction, *F*(9, 114) = 2.50, *p* = 0.012, *ηp*^2^ = 0.13. Examination of effect sizes indicates that while the largest effect on latency is attributable to differences across days, both condition and the interaction between condition and day represent medium-sized effects. Paired *t*-tests with a Bonferroni correction were used to analyze the nature of the main effect of day and revealed that mean latency decreased significantly between each of the days except for between days 3 and 4, *p* < 0.001 for all significant comparisons, day 1 (*M* = 40.19, *SD* = 10.54); day 2 (*M* = 20.08, *SD* = 9.26); day 3 (*M* = 11.59, *SD* = 5.48); day 4 (*M* = 11.01, *SD* = 7.06). The nature of the main effect of Condition was examined by Tukey’s Honestly Significant Difference (HSD) test, which determined that LMA-Controls performed marginally worse than EMA-Controls (*M* = 19.25, *SD* = 1.59, *p* = 0.078), confirming that older rats on the control diet were beginning to show signs of cognitive decline. Moreover, LMA rats on the control diet performed significantly worse (*M* = 24.72, *SD* = 1.52) than LMA rats on the HAD diet (*M* = 18.88, *SD* = 1.52; *p* = 0.045), demonstrating that the HAD diet had a beneficial effect on the older animals’ performance. 

**Figure 1:**
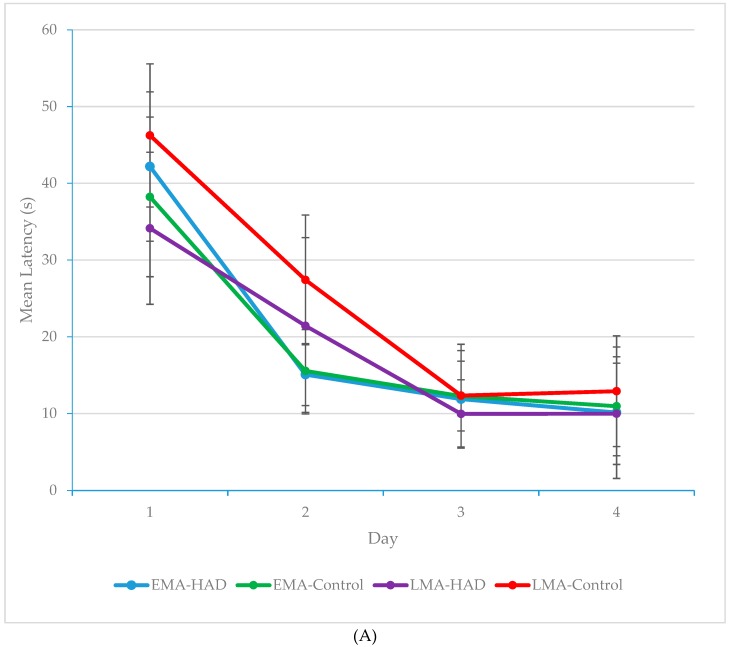

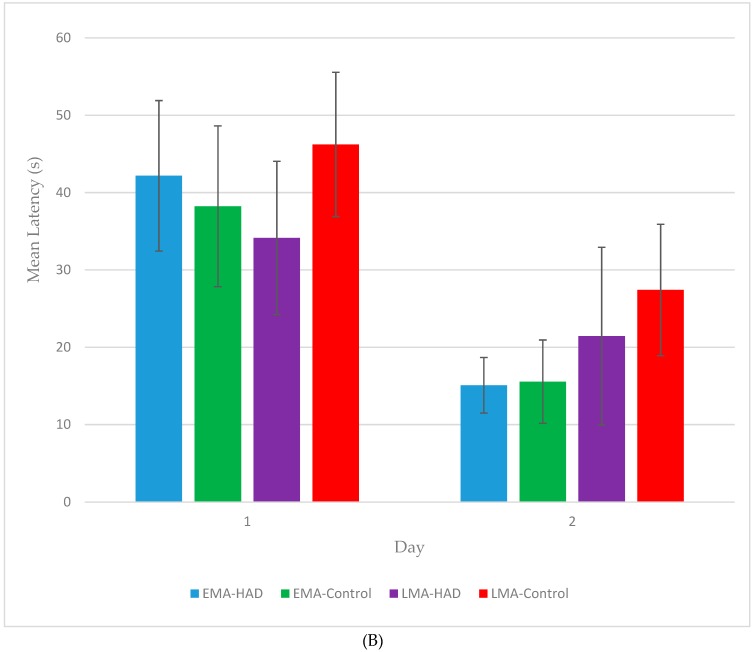
(**A**) Mean latency in seconds (s) for each condition on each training day (collapsed across the four daily trials). LMA-HAD (Late middle-age-high antioxidant diet) rats performed as well on days 1 & 2 as EMA rats, whereas LMA-Controls lagged behind until day 3. Error bars represent Standard Deviation (SD). (**B**) Mean latency in seconds (s) for each condition on training days 1 & 2 (collapsed across the four daily trials). LMA-HAD rats performed as well on days 1 & 2 as early middle age rats, whereas LMA-Controls lagged behind until Day 3. Error bars represent SD.

The nature of the interaction effect, which was of principal interest, was explored using simple main effects analyses. Between-condition differences for each day were examined using one-way ANOVAs and post-hoc Tukey’s tests. As can be seen from Figure 1A, mean latency differed by Condition on days 1 (*F*(3, 41) = 3.04, *p* = 0.041, *ηp*^2^ = 0.19) and 2 (*F*(3, 41) = 5.52, *p* = 0.003, *ηp*^2^ = 0.30), but not 3 and 4 (*p* > 0.05). It can be seen from Figure 1B that specifically, LMA-Controls performed significantly worse (*M* = 46.23, *SD* = 9.33) than LMA-HAD animals on Day 1 (*M* = 34.14, *SD* = 9.91; *MD* = 12.09, *p* = 0.031), while all other conditions performed similarly. On Day 2, the LMA-Controls performed more poorly (*M* = 27.41, *SD* = 8.5) than the EMA-Controls (*M* = 15.55, *SD* = 5.39; *MD* = 11.86, *p* = 0.009) and the EMA-HAD animals (*M* = 14.39, *SD* = 3.6; *MD* = 12.33, *p* = 0.006). Conversely, the LMA-HAD animals (*M* = 21.43, *SD* = 11.49) performed similarly to both EMA conditions (*p* > 0.05). Thus, rats in all conditions learned to successfully find the submerged platform by Day 3, but rats in the LMA-HAD condition performed on a par with the EMA conditions a day earlier in training than did the LMA-Controls. Examination of *ηp*^2^ values indicates that the effect of condition was largest between groups on day 2 with 30% of the variability in latency being explained by condition. This represents a strong effect. It is in some ways not surprising that the largest between-condition effect is seen on day 2 given that performance in the water maze on day 1 is poor in all groups since they’ve had very limited exposure and opportunity to learn the spatial layout of the maze. Thus any improvement in learning and memory functioning conferred by the diet should be more clearly expressed on day 2. These results demonstrate the ability of an eight-week, whole foods HAD to preserve the learning curve in aging female rats to match that of younger rats on a hippocampal-dependent learning and memory task. 

#### Path Length

A 4 × 4 mixed design ANOVA examining mean path length in centimeters (converted from inches) by day and condition revealed a pattern of results very similar to latency, revealing significant main effects of day, *F*(3, 114) = 120.75, *p* = 0.000, *ηp*^2^ = 0.76, and condition, *F*(3, 38) = 3.25, *p* = 0.032, *ηp*^2^ = 0.20, and a significant interaction, *F*(9, 114) = 2.23, *p* = 0.034, *ηp*^2^ = 0.15. Examination of effect sizes again indicates that while the largest effect on path length is attributable to differences across days, both condition and the interaction between condition and day represent medium-sized effects. Paired *t*-tests with a Bonferroni correction were used to analyze the nature of the main effect of day and revealed that mean latency decreased significantly between each of the days except for between days 3 and 4, *p* < 0.001 for all significant comparisons, day 1 (*M* = 1138.96, *SD* = 314.40); day 2 (*M* = 619.43, *SD* = 301.71); day 3 (*M* = 353.36, *SD* = 171.86); day 4 (*M* = 305.92, *SD* = 189.79). The nature of the main effect of condition was examined by Tukey HSD test, which determined that LMA-Controls performed significantly worse (*M* = 716.89, *SD* = 42.19) than LMA rats on the HAD (*M* = 555, *SD* = 42.19, *p* = 0.047), confirming that older rats on the HAD outperformed their standard diet counterparts. 

**Figure 2:**
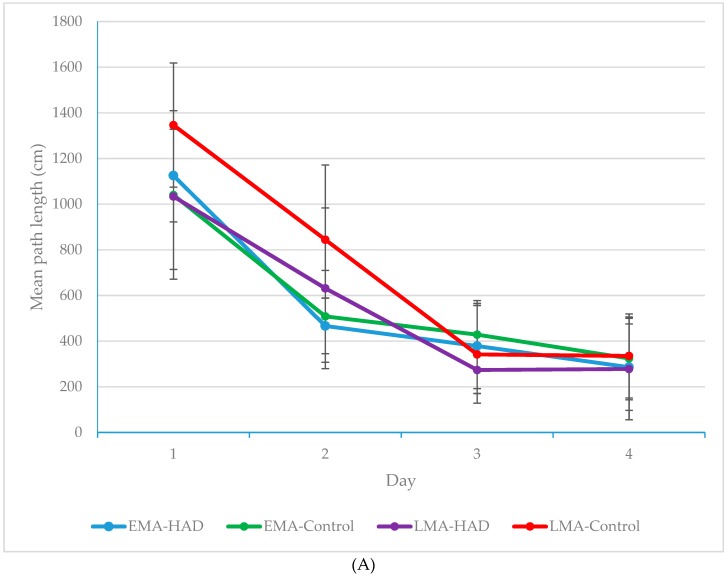

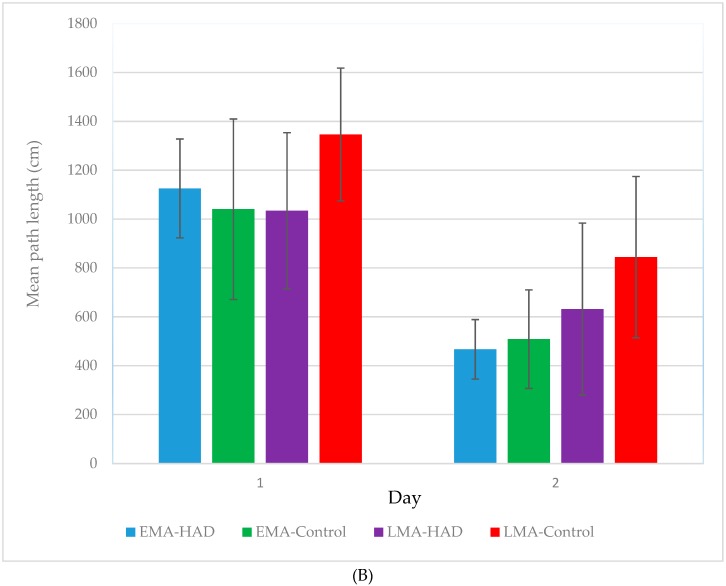
(**A**) Mean path length in centimeters (cm) for each condition on each training day (collapsed across the four daily trials). LMA-HAD rats performed as well on days 1 & 2 as EMA rats, whereas LMA-Controls lagged behind until day 3. Error bars represent SD. (**B**) Mean path length in centimeters (cm) for each condition on training days 1 & 2 (collapsed across the four daily trials). LMA-HAD rats performed as well on days 1 & 2 as EMA rats, whereas LMA-Controls lagged behind until day 3. Error bars represent SD.

Although the main effects were in-line with our hypotheses, it was the nature of the interaction effect that was of principal interest. The interaction effect was explored using simple main effects analyses. Between-condition differences for each day were analyzed using one-way ANOVAs and post-hoc Tukey’s tests. As can be seen in Figure 2A, mean path length was marginally different by

Condition on day 1 (*F*(3, 41) = 2.62, *p* = 0.065, *ηp*^2^ = 0.17) and significantly different on day 2 (*F*(3, 41) = 4.12, *p* = 0.013, *ηp*^2^ = 0.25). Conditions did not differ on days 3 or 4 (*p* > 0.05). As was true for latency, and likely for the same reasons, *ηp*^2^ values show that the effect of condition was largest between groups on day 2 with 25% of the variability in path length being explained by condition. Figure 2B shows what post-hoc tests revealed, which is that on day 1, LMA-Controls performed marginally worse (*M* = 1346.33, *SD* = 271.91) than EMA-Controls (*M* = 1040.38, *SD* = 369.32, *MD* = 305.92 *p* = 0.10), mirroring latency findings which showed that older animals were somewhat retarded in their ability to navigate to the platform compared to their younger counterparts early in training. Moreover, the HAD diet had a beneficial effect on the performance of the older rats, as animals in the HAD condition had a marginally shorter average path length on day 1 (*M* = 1040.38, *SD* = 369.32) than rats in the control diet condition (*MD* = −312.62, *p* = 0.082). On day 2, the LMA-Controls performed significantly more poorly (*M* = 844.27, *SD* = 327.53) than either of the younger groups (EMA-Controls, *M* = 508.51, *SD* = 201.45; *MD* = −335.74, *p* = 0.036; EMA-HAD, *M* = 466.60, *SD* = 121.87; *MD* = 377.67, *p* = 0.015). The LMA-HAD, by contrast performed on a par with the younger groups. By Days 3 and 4, rats in all conditions had learned to successfully navigate to the hidden platform. 

These data very closely align with the latency data by showing superior performance in the LMA-HAD group early in training, especially on day 2, compared to their standard diet counterparts. Moreover, the agreement of the path length and latency data suggest that the shorter mean latencies in the LMA-HAD group early in training were due to better memory for the platform location rather than a nonspecific effect of the HAD, such as motivation. 

Probe trial. There were no between-condition differences in either total time or percent time spent in the target quadrant (SW) on the probe test (*p* > 0.05). This is an expected finding given that the probe trial took place at the very end of training on Day 4 once between-group differences on the other dependent measures had disappeared and all treatment groups had learned the spatial location of the hidden platform based upon the extra-maze visual cues.

Mean Speed (cm/s). Because of the possibility that HAD could have improved latency scores in the older rats by mitigating an age-related loss of fitness rather than by improving memory for the platform location, we compared the mean speed of rats (cm/s) by day and condition using a 4 × 4 mixed design ANOVA. Mean speeds are reported in Table 1. There was a significant main effect of Day, *F*(3, 114) = 4.14, *p* = 0.012, *ηp*^2^ = 0.098, however, paired t-tests with a Bonferroni correction revealed no significant between-day differences (*p* > 0.01). There was also a significant main effect of Condition, *F*(3, 38) = 4.90, *p* = 0.006, *ηp*^2^ = 0.28, and a significant interaction, *F*(9, 114) = 2.33, *p* = 0.028, *ηp*^2^ = 0.15. Examination of *ηp*^2^ reveals that although the main effect of day was significant, the effect size was small by comparison to the effects of both condition and the interaction. A Tukey’s HSD test showed that Group EMA-Control swam significantly faster, on average (*M* = 28.83, *SE* = 1.07), than either Group LMA-Control (*M* = 24.41, *SEM* = 1.02, *MD* = 4.45, *p* = 0.02) or LMA-HAD (*M* = 24.59, *SEM* = 1.02, *MD* = 4.24, *p* = 0.03). 

The interaction effect, which was of principle interest, was analyzed using simple main effects analyses. Between-condition differences in speed for each day were analyzed using one-way ANOVAs and post-hoc Tukey’s tests. Table 1 shows mean speed by condition across each of the four training days. Mean speed differed by condition on days 2, 3, and 4 (*F*(3, 38) = 3.45, *p* = 0.026, *ηp*^2^ = 0.21; *F*(3, 38) = 6.48, *p* = 0.001, *ηp*^2^ = 0.34; and *F*(3, 38) = 4.86, *p* = 0.006, *ηp*^2^ = 0.28, respectively). Since there were only differences in latency and path length on days 1 and 2 and since speed was not different by condition on day 1, we will only report post-hoc results for Day 2. On the second day of training, EMA-Controls swam marginally faster (*M =* 30.25, *SD* = 5.28) than LMA-Controls (*M =* 25.21, *SD* = 3.88; *MD* = 5.05, *p* = 0.057) and significantly faster than the LMA-HAD group (*M = 25.1, SD* = 3.76; *MD* = 5.16, *p* = 0.05). This is an interesting and important finding because it demonstrates that despite swimming more slowly than the younger groups on day 2, Group LMA-HAD performed on a par with them in regards to latency and path length. It is also important to note that at no time point did the swimming speed of the older groups significantly differ from one another. In other words, superior performance in regards to latency and path length by the HAD group cannot be attributed to superior swimming speed in that group compared to the old, standard diet group. These findings support the interpretation that the HAD conferred its beneficial impact via memory and cognitive effects rather than on fitness or other nonspecific motor or motivational effects. Moreover, as is evident from Table 1, both late middle age groups swam more slowly (although not necessarily significantly so) than both early middle age groups each day. That finding makes it very important that our path length data mirror our latency data, since latencies could have been affected by swimming speed, whereas path length would not.

## 4. Discussion

The pattern of results from the latency and path length analyses were complimentary and showed that although rats in all conditions learned to locate the hidden platform across training days, the HAD conferred an advantage to older animals, allowing them to perform as well as their younger counterparts by day 2, rather than day 3. Speed data showed that older rats swam slightly, but significantly slower than younger rats, thus the path length data are of special importance since they are not confounded by swimming speed. Importantly, swimming speed did not differ between the older groups at any time point, so it cannot account for the different performance between older groups in terms of latency. 

Consistent with past research in male rats showing that strawberry or spinach extracts were able to prevent the decline in MWM performance typically seen in aging organisms [28], our results showed that a diet supplemented by freeze-dried spinach and strawberries was capable of delaying the small, but measureable decrement in spatial learning that occurred in intact (non-ovariectomized) female rats as they entered late middle age. Given that nearly two-thirds of Americans currently living with AD are women [37] (p. 19), we believe our results offer an important extension of past findings. Another advantage of our design is that the improvement in cognitive performance was produced using a real foods dietary supplement of short duration, indicating that megadoses of antioxidants over long periods of time, which may be costly and have undesirable side effects [33], are not necessary to realize cognitive benefits at this stage of the lifespan in female rats. Further research will be needed to determine if these procognitive effects generalize to other foods with different antioxidant profiles. Strawberries and spinach are high in flavonoids, among other antioxidants, and recent research suggests that flavonoids are particularly effective in alleviating cognitive deficits associated with aging due to their ability to reduce oxidative stress and inflammation in the brain while increasing neuroplasticity [25]. Thus other high flavonoid foods might also be good candidates for dietary intervention at this stage of the lifespan in females; however, experimental tests are needed to confirm that hypothesis. 

Although our study was behavioral in nature and not designed to determine mechanism, it is interesting to note that researchers have discovered a number of possible means by which antioxidants may exert their beneficial effects on cognitive function, including both classical antioxidant activity, such as via free-radical scavengers that combat oxidative stress [25], as well as by other mechanisms. In an early study, researchers found that age-related losses in the ability of G proteins to respond quickly to receptor activation (a process that initiates the cellular cascade of events leading to long-term memory formation) was reversed by a diet rich in Vitamins E and C [27]. In that same study, the authors reported that the antioxidant-rich diet also stalled age-related decline in calcium uptake that is required for normal neurotransmitter regulation [27]. Moreover, it has been reported that higher levels of α-tocopherol in the hippocampuses of old rats that had been fed a diet supplemented with strawberry, blueberry, or spinach extracts and higher levels of γ-tocopherol in rats fed strawberry extracts [28]. These brain changes were accompanied by superior learning curves compared to rats fed a control diet [28]. In a 2008 review [24], several intriguing mechanisms beyond classical antioxidant activity were explicated. According to the authors, antioxidants (flavonoids, in particular) may exert their neuroprotective effects by “(1) the modulation of intracellular signalling cascades which control neuronal survival, death and differentiation; (2) affecting gene expression and (3) interactions with mitochondria” [24] (p. 60). Moreover, improved neuroplasticity, increased cortical blood flow (which may improve learning and memory by facilitating neurogenesis in the hippocampus), and inhibition of neuroinflammation are also likely mechanisms [25,38].

Large-scale epidemiological, as well as short-duration human experimental studies of the cognition-sparing effects of dietary antioxidants in aging have produced mixed results [34,38,39,40]. This is not surprising given the difficulty of isolating the effects of specific nutrients in epidemiological studies. Moreover, as aptly noted by the authors of a 2013 review, inconsistent outcomes in experimental studies may be due to “large heterogeneity in study design, differential control of confounders, insufficient measures of cognitive performance, and difficulties associated with dietary assessment” [39] (p. 279). While a 2013 review of population-based cohort studies found positive effects of vitamins C and E, as well as carotenoids and flavonoids in several studies deemed to be of high or adequate quality [40], a 2013 meta-analysis of 21 studies reported mixed findings [39]. A more recent review [38] presented numerous epidemiological studies showing positive effects of long-term flavonoid intake on cognitive function in healthy adults. We hope that the strong support for the beneficial effects of antioxidants that has been generated by experimental work with animals, including the current study, combined with the promising results of human research will encourage the undertaking of human clinical trials.

## Figures and Tables

**Table 1:** Mean speed ^1^ (standard deviation in parentheses) across training days by condition.

Group	*n*	Day			
		1	2	3	4
EMA-Control	10	28.02(3.68)	30.26(5.28)	29.52(4.63)	27.53(4.59)
EMA-HAD	10	27.16(3.48)	28.40(4.61)	28.08(3.54)	28.36(4.35)
LMA-Control	11	25.57(2.88)	25.21(3.88)	23.63(3.38)	23.18(2.71)
LMA-HAD	11	26.66(2.79)	25.10(3.76)	23.48(4.06)	23.14(4.47)

^1^ Speed reported in cm/s; EMA: Early Middle Age; HAD: High Antioxidant Diet; LMA: Late Middle Age.
